# Posterior Polar Annular Choroidal Dystrophy: Genetic Insights and Differential Diagnosis in Inherited Retinal Diseases

**DOI:** 10.3390/cimb46020089

**Published:** 2024-02-05

**Authors:** Francesco Ruggeri, Chiara Ciancimino, Antonio Guillot, Daniele Fumi, Federico Di Tizio, Serena Fragiotta, Solmaz Abdolrahimzadeh

**Affiliations:** 1 Ophthalmology Unit, Neurosciences, Mental Health, and Sense Organs (NESMOS) Department, Faculty of Medicine and Psychology, University of Rome Sapienza, 00185 Roma, Italy; fra.ruggeri@uniroma1.it (F.R.); chiara.ciancimino@uniroma1.it (C.C.); antonio.guillot@uniroma1.it (A.G.); daniele.fumi@uniroma1.it (D.F.); 2 St. Andrea Hospital, Via di Grottarossa 1035/1039, 00189 Rome, Italy; fditizio@ospedalesantandrea.it; 3 UOC Ophthalmology, Department of Surgical Areas, S.M. Goretti Hospital, 04100 Latina, Italy; s.fragiotta@ausl.latina.it

**Keywords:** posterior polar annular choroidal dystrophy, multimodal imaging, spectral domain optic coherence tomography, retinal pigment epithelium, inherited retinal diseases, choroideremia, retinitis pigmentosa, gyrate atrophy of retina and choroid, central areolar choroidal dystrophy

## Abstract

Posterior polar annular choroidal dystrophy (PPACD) is a rare ocular disorder and presents as symmetric degeneration of the retinal pigment epithelium (RPE) and the underlying choriocapillaris, encircling the retinal vascular arcades and optic disc. This condition distinctively preserves the foveal region, optic disc, and the outermost regions of the retina. Despite its distinct clinical presentation, due to the infrequency of its occurrence and the limited number of reported cases, the pathophysiology, and the genetic foundations of PPACD are still largely uncharted. This review aims to bridge this knowledge gap by investigating potential genetic contributors to PPACD, assessing current findings, and identifying genes that warrant further study. Emphasis is also placed on the crucial role of multimodal imaging in diagnosing PPACD, highlighting its importance in understanding disease pathophysiology. By analyzing existing case reports and drawing comparisons with similar retinal disorders, this paper endeavors to delineate the possible genetic correlations in PPACD, providing a foundation for future genetic research and the development of targeted diagnostic strategies.

## 1. Introduction

First documented in 2010, posterior polar annular choroidal dystrophy (PPACD) is a rare disease with bilateral involvement prevalently characterized by atrophy of the retinal pigment epithelium (RPE) and choriocapillaris configuring a peculiar annular pattern surrounding the peripapillary region extending along the temporal vascular arcades, preferentially leaving intact the peripheral retina, foveal zone, and optic disc [1,2,3,4]. Individuals affected presented a variable age of presentation comprised between 30–68 years, in almost all the cases described, except for a single case of a young boy of unspecified age [1,2,3,5,6,7]. In the initial stages, the visual acuity can be preserved or slightly reduced, with patients complaining of night blindness [1,7]. Typically asymmetric in its bilateral presentation, since its first identification, few cases have been reported, of whom only one presented foveal involvement with cystoid macular edema in both eyes [5]. A possible variant of the disease known as posterior polar hemispheric choroidal dystrophy (PPHCD) is characterized by similar atrophic lesions spreading along a single retinal vascular arcade following a hemispheric configuration [3]. Long-term data are limited with only two cases reporting a follow-up with the patient for over five years [2,8]. The case reported by Sone and colleagues showed progression of fundus lesions and consequent visual field deterioration suggesting the possible progressive nature of the disease [2]. Bommakanti and colleagues reported a PPACD case followed up for over 5 years. During this period, the patient developed posterior subcapsular cataract, cystoid macular edema, and retinal neovascularization [8]. This instance indicates that the progression of PPACD might comprehend complications common to various retinal dystrophies, highlighting the need for diligent and longer follow-up and potentially revising prognostic expectations for this condition [2,8].

Case reports currently available in the literature share a common clinical presentation, with nyctalopia usually reported as the first symptom affecting patients’ quality of life with individuals experiencing difficulty seeing in low-light or dark conditions [1,2,3,5,6,7]. As the disease progresses, patients experience peripheral visual loss with arcuate scotomas on visual field testing corresponding to chorioretinal atrophy areas identified at dilated fundus examination [1]. Cases described in the literature share a unique clinical appearance. However, an exhaustive multimodal imaging approach remains crucial to diagnose PPACD, including fluorescein angiography (FA), spectral domain optical coherence tomography (SDOCT), fundus autofluorescence (FAF), Humphrey visual field testing, and electroretinography (ERG) [1,2,3,5,6].

Genetic testing has been investigated in only two case reports [3,5]. In the case of a patient of African American descent, several gene mutations of uncertain significance were identified. Many of these mutations fall into genetic pathways implicated in retinal functioning, particularly the NR2E3 gene that has been implicated in several inherited retinal dystrophies [3].

**Figure 1:**
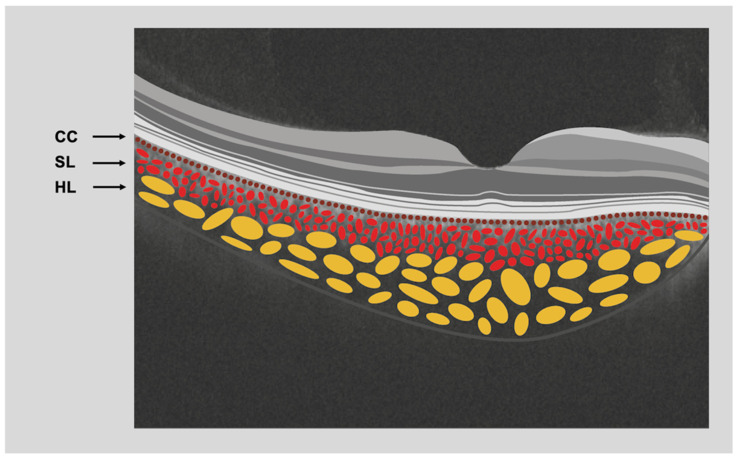
Schematic view of the choroid. Arrows show the choroidal vessel layers; choriocapillaris (CC), Sattler’s layer (SL) and Haller’s layer (HL) (graphics courtesy of Dariush Rahimi).

Clinical and multimodal findings and symptoms referred to by PPACD patients should guide further genetic investigations. For instance, nyctalopia has been observed in most cases, suggesting a potential impact on the rod photoreceptors, which are predominantly for vision in dim light. In PPACD, impairment of choriocapillaris and RPE in a characteristic topographical distribution may contribute to the primarily affected rods [3,6]. The exact mechanism by which PPACD contributes to nyctalopia remains to be elucidated. However, it has been hypothesized that the choroidal impairment affecting the outer retina perfusion may interfere with the phototransduction cascade of the rods, leading to impaired scotopic vision [3,6]. A schematic representation of choroidal layers can be seen in Figure 1. 

The interplay of genetic factors in retinal diseases is highlighted by research demonstrating the involvement of genes like VEGF-A and the Otx family in the pathology of proliferative vitreoretinopathy (PVR) [9]. While PVR is distinct from PPACD, the shared genetic expressions suggest underlying molecular pathways that could influence retinal disease progression and potential therapeutic strategies for conditions like PPACD [9].

The present review aims to offer a comprehensive understanding of the prevalence, clinical signatures, and multimodal findings defining typical PPACD. Furthermore, clinical features and genetic findings are interpreted in comparison with similar clinical conditions aiming to elucidate the clinical spectrum of the disease.

## 2. PPACD Diagnostic Approaches

### 2.1. Fundus Examination

Since its first documentation by Yannuzzi and colleagues in 2010 the funduscopic appearance presented peculiar morphological phenotypical characteristics [4]. Characteristically, peripapillary chorioretinal atrophy spreads toward the temporal vascular arcades, configuring an annular pattern with foveal sparing. Lesions are often asymmetric between eyes, with one eye that is more severely involved. In one case, Lenis and colleagues reported that a symmetric atrophy was noted in both eyes [10]. Although discrete pigment aggregations can be observed within the atrophic regions, the peripheral retina is devoid of bone spicule-like pigment alterations and arteriolar narrowing, distinguishing this entity from other inherited retinal dystrophies [1,2,5,6,10].

### 2.2. Fundus Autofluorescence

FAF has emerged as a pivotal noninvasive technique for various retinal disorders, especially in the spectrum of retinal and choroidal dystrophies. FAF imaging is based on the caption of light emitted from lipofuscin in the RPE, where an increased autofluorescence typically signifies an accumulation of fluorophores within the RPE [11].

In PPACD, short wavelength FAF shows an annular pattern of hypoautofluorescence in a peripapillary distribution, extending concentrically towards the retinal vascular arcades corresponding to the chorioretinal atrophy. A perifoveal hyperautofluorescent border can be frequently observed and demarcates the transition from healthy to atrophic retinal tissue [1,2,3,5,10].

### 2.3. Fluorescein Angiography

FA typically shows a window defect in the atrophic area with visualization of the underlying choroidal vasculature attributable to choriocapillaris and RPE loss. Moreover, a pattern of concentric staining in the late frames around the optic disk and central macula can be appreciated [3,5,6,10]. Tabbaa and colleagues demonstrated areas of late leakage surrounding the fovea in one case with PPHCD [3]. 

### 2.4. Optical Coherence Tomography

SD-OCT typically reveals the reduced thickness of the outer retina and the choriocapillaris colocalizing with the regions exhibiting the atrophic changes on clinical examination, while the remaining retina appears intact. Additionally, disturbances in the organization of the outer retina and alterations in the ellipsoid zone have been observed [5]. Furthermore, the concomitance of intraretinal cystic changes was noted in two distinct cases, with parafoveal localization in one case [3] and bilateral central cystoid changes in the other case [5]. 

Swept-source optical coherence tomography (SS-OCT) represents an enhancement in imaging capabilities, offering superior resolution and deeper tissue penetration [12,13]. SS-OCT confirmed the presence of retinal thinning, rarefaction of the ellipsoid band, and RPE with choriocapillaris attenuation [1,2,6]. Sone et al. [2] also noticed a dilation of the Sattler and Haller’s choroidal vascular layers. The enface maps segmented on RPE further demonstrated the dilation of these vascular layers. However, Narayan [6] reported a reduction of choroidal thickness in the areas involved with a reduced Sattler’s layer.

Of note, despite OCT B scans enabling high-resolution cross-sectional visualization of the retina, it is crucial to recognize the distribution of the lesions in PPACD. Indeed, the predilection of PPACD for the mid- and peripheral retina [1,2,3,5,6,7] necessitates expanding the scan area to capture the pathological features. In this regard, wide-field OCT may facilitate a more precise monitoring of disease progression. By documenting changes in retinal thickness and structure in a broader area, the peripheral spread of the dystrophy can be tracked with greater accuracy. This information is vital for prognostic assessment and for tailoring, monitoring, and management strategies to the individual patient’s needs [14].

### 2.5. Optical Coherence Tomography Angiography

OCTA showed a preservation of the central superficial vascular plexus. Conversely, a notable reduction in flow signal was noted in the deep capillary plexus, accompanied by a significant rarefaction of the choriocapillaris corresponding to the affected areas [5,6]. The reduction of vascular flow on OCTA was hypothesized to be secondary to outer retina atrophy, thus confirming that the disease can be in the dystrophic spectrum [6].

### 2.6. Perimetric Testing

Perimetric testing performed in cases of PPACD [2,3] showed superior arcuate scotomas in both eyes, aligning with the atrophic regions observable upon funduscopic examination. In the two cases reported by Lenis and colleagues, the authors reported enlarged blind spots in perimetric testing correlating with the pattern of peripapillary atrophy [10]. Others [1,6] demonstrated generalized depressed points, predominantly in the pericentral area [1].

### 2.7. Full-Field Electroretinography

Full-field ERG in PPACD typically shows predominately diffused depressed responses, not solely restricted to the atrophic regions [3,6,8]. However, there is a diminished response in both scotopic and photopic a-wave and b-wave amplitudes, alongside a decrease in the 30 Hz flicker response, and oscillatory potentials with a prolongation of implicit time [1,6]. In the full-field ERG analysis, a mild decrease in the a-wave amplitudes under scotopic conditions and combined rod–cone responses were observed for both eyes. The cone and 30 Hz flicker responses appeared within the normal range but were slightly reduced [2]. In a documented case by Del Valle et al., full-field ERG demonstrated diminished cone function while rod function remained within normal limits, suggesting widespread cone dysfunction [5]. This finding stands in contrast to other observed results.

### 2.8. Laser Speckle Flowgraphy

Laser speckle flowgraphy (LSFG) is a dye-free imaging mode that utilizes the statistical analysis of laser speckle patterns to evaluate ocular blood flow. This technique has been demonstrated to be useful in assessing circulatory dynamics within the retina, optic nerve head (ONH), and choroidal vessels [15]. LSFG was conducted in only one case with PPACD revealing a cold spectrum in the color range of both eyes [2]. A summary of PPACD appearance with different imaging modalities can se seen in Table 1. 

## 3. Genetic Basis of PPACD

The scarcity of literature addressing the casuistry associated with PPACD is evident in the limited utilization of genetic screenings in affected individuals. Specifically, there exist only two case reports wherein potential genetic correlations were systematically assessed, focusing on distinct genetic loci. In the case documented by Lenis et al. [10], no substantial mutations were documented after testing genes such as ABCA4, BEST1, PRPH2, CDH3, EFEMP1, ELOVL4, IMPG1, IMPG2, PROM1, RDS, and TIMP3. Furthermore, no analogous clinical or semiotic aberrations were observed in first-degree relatives. In a second case suffering from PPHCD [3], a standardized SPARK Inherited Retinal Dystrophy (IRD) panel was performed testing several mutations of indeterminate significance across multiple genes, including CCD2D2A, CEP78, NR2E3, PCARE, PEX14, and RPGRIP1. Unfortunately, the utility of identifying these mutations is constrained by various factors, with the primary challenge being the unique nature of the sample in which their presence is evident. The establishment of clear connections between specific gene mutations (genotype) and disease characteristics (phenotype) is thus, hindered. Additionally, it is crucial to recognize that many of the implicated genes lack a fully defined role within the retina, leading to significant gaps in certain phenotype–genotype correlations.

In this context, Next-generation Sequencing (NGS) could be pivotal, offering detailed insights into the genetic landscape of PPACD by identifying both prevalent and rare variants that might be implicated in its pathogenesis [16].

Nevertheless, NGS presents challenges, including the complex analysis of vast genomic data sets and distinguishing between pathogenic mutations and benign variants. The integration of NGS into PPACD research holds promise for uncovering new genetic targets and enhancing personalized medicine approaches. However, this requires the collaborative effort of experts in genomics, bioinformatics, and ophthalmology to fully interpret the intricate data NGS provides, all within the clinical context of PPACD [16].

Notably, a substantial proportion of the mutations under scrutiny are associated with cone–rod dystrophy, implicating a common pathogenesis that specifically affects photoreceptors [3]. The potential genetic involvement in PPACD is encouraged by characteristic features, such as night blindness, ERG alterations, and visual field defects.

Sharon and colleagues indicate that the RPE allocates over 9.5% of its transcripts to the synthesis of proteins involved in this degradation process, a stark contrast to the neural retina, which dedicates less than 3% [17]. This disparity underscores the specialized function of the RPE in maintaining photoreceptor health and potentially implicates the degradation pathway in the pathology of retinal diseases like PPACD [17]. Given the essential functions of the RPE in photoreceptor maintenance and the nascent understanding of its genetic landscape, it becomes increasingly important to explore how aberrations in these genes might contribute to retinal dystrophies. In particular, the identification of pathologic mutations within RPE-related genes may offer valuable insights into the molecular underpinnings of PPACD. This understanding is crucial not only for the diagnosis and prognosis of the disease but also for the development of targeted therapies that address the underlying genetic defects [17].

In this section, the genes potentially involved in PPACD and their role in maintaining retinal homeostasis are discussed.

A case report found a PPHCD patient positive for a partial NR2E3 deletion demonstrating a role of this gene in the phenotypic manifestations of the pathology [3].

The nuclear receptor NR2E3, commonly known as the photoreceptor-specific nuclear receptor (PNR), is a transcriptional regulator localized to photoreceptors in both the developing and adult retina and is part of the extensive nuclear hormone receptor superfamily [18,19]. Conditions associated with mutations in NR2E3 are marked by a reduced population of rod photoreceptors alongside an increase in cells resembling short-wavelength sensitive cones [19]. Researchers have discovered thirty-two distinct mutations in the NR2E3 gene. Clinically, mutations in the NR2E3 gene manifest in several IRDs sharing common symptoms such as early-onset nyctalopia and minimal rod cell functionality, phenotypic characteristics typically found in PPACD [18,20].

NR2E3 is part of the nuclear receptor family, comprising 48 members, including endocrine and orphan receptors [21]. During the embryonic stages, both rod and cone cells originate from a shared precursor specific to photoreceptors [21,22]. The NR2E3 gene plays a pivotal role in guiding the differentiation toward the rod cells rather than the cones. Investigations involving both animal models and humans with NR2E3 mutations indicate that this gene has a dual function in photoreceptor differentiation: it inhibits the expression of genes associated with cones, e.g., OPNSW1 (encoding blue opsin), GNAT2, and the cone transducin subunits, while simultaneously facilitating the activation of genes specific to rods, including GNB1 (the rod transducin β subunit) and rhodopsin [21].

In the genetic analysis performed in the case of PPHCD, different variants of uncertain significance emerged, including CC2D2A, CEP78, NR2E3, PCARE, PEX14, and RPGRIP1 [3].

PCARE is postulated to be critical in the early stages of outer segment (OS) disk formation, orchestrating the relocation of actin-associated elements to the photoreceptor OS foundation. Rod photoreceptors undergo a daily renewal process where approximately 10% of their opsin-rich disks at the apical tip of the OS are shed and subsequently engulfed by neighboring RPE cells. The genesis of new photoreceptor disks commences at the site where the connecting cilium (CC) adjoins the OS base, marked by an outpouching of the ciliary plasma membrane. The protein PCARE is known to predominantly interact with WASF3, which influences the actin scaffolding within the cell by stimulating the ARP2/3 complex. This stimulation is a precursor to the formation of a branched network of F-actin from G-actin, an essential structure for the ciliary membrane to protrude, facilitating the enlargement of the ciliary apex that drives disk morphogenesis. Clinically, patients often exhibit initial deterioration in the cone photoreceptor system, evident through macular abnormalities and annular scotomas during visual field evaluation [23].

RPGRIP1 encodes the retinitis pigmentosa (RP) GTPase regulator interaction protein. It is situated on chromosome 14 (14q11.2) and spans 63 kb, comprising 24 exons that translate into a protein consisting of 1286 amino acids [24,25]. The gene RPGRIP1 synthesizes the protein known as the RP GTPase regulator interaction protein plays a fundamental role in the functions of the retina. Its activity is aligned with that of its molecular associate, the RPGR, at the OS of photoreceptors in humans. RPGRIP1 plays a vital role in protein transport regulation from the inner to OS of these cells [24,25,26]. Experimental models in mice have illustrated the essential role of RPGRIP1 in the development of photoreceptor disk morphology; photoreceptor degeneration occurs despite the normal initial formation of rods and cones [24,25]. The Human Gene Mutation Database (HGMD) documents various pathogenic mutations in RPGRIP1, such as missense, splicing, deletion, duplication, and frameshift variations. Moreover, infrequent structural variations and complex mutations, including noncoding mutations like a homozygous deletion within exon 17 of RPGRIP1, have been identified. These findings suggest that complex and noncoding mutations could substantially contribute to the overall mutational landscape of the gene [24,25,27]. A summary of retinal genes and their function can be found in Table 2.

## 4. Differential Diagnoses and Their Genetic Patterns

Several inherited retinal conditions share similar clinical characteristics with PPACD and therefore should be ruled out when making a diagnosis of PPACD. The knowledge of the inherited retinal disorders with a similar clinical phenotype may help clarify the possible genes involved in order to address future genetic research for this condition.

### 4.1. Diagnostic Differences between Retinitis Pigmentosa and PPACD

Inherited retinal disorders impact over 200,000 individuals, as reported in a study in the United States, with a global impact reaching the millions of people affected. RP is responsible for about half of these cases. This condition is characterized by heterogeneous clinical features, including variability in the age of onset, progression speed, the involvement ratio of rod-to-cone photoreceptors, and the extent of RPE involvement [19,54,55].

Clinically, RP typically presents a classic triad of signs: typical waxy pallor of the optic disc, pigmentation with bone spicule morphology, and attenuation of retinal vessels. Diminished or absent ERG responses and compromised perimetric testing are other features. Nevertheless, clinical phenotypes can vary owing to multiple gene involvement, where each gene can have several alleles [54,56,57]. The clinical triad of RP can resemble PPACD presentation, particularly in the presence of pigmentary changes and the annular distribution [1,2,3,5,6]. However, distinguishing features include the absence of bone spicule-like lesions, normal retinal vasculature, and optic disc appearance.

Among RP heterogeneous phenotypic manifestations, sector RP represents a variant initially reported by Bietti [58], which can also share similarities with PPACD and should be taken into consideration in the diagnostic process, particularly in the hemispheric variant of PPACD. As suggested by Narayanan in 2018, hemispheric and annular dystrophy could be part of the same spectrum of disease attributable to PPACD [6].

Sector RP is characterized by symmetrical retinal degeneration confined to a single quadrant or retinal hemifield. Sector RP accounts for approximately 2% of all RP subtypes, with a steady or slow progression compared to the progression of classic RP [55,59]. As mentioned above, distinctive clinical features include optic disc pallor, bone-spicule pigmentation, and attenuation of retinal vessels, which have not yet been described in PPACD. However, it is worth noticing that the manifestation of bone spicules is not uniform across all individuals with RP; some may exhibit fine, dust-like deposits, while others may present with coin-shaped areas of increased pigmentation [54,57,59].

Regarding the visual field defects revealed through perimetry, a pattern of symmetrical visual field reduction is common in sector RP, often initiating as discrete spots of loss in the mid-peripheral vision. Over time, these spots tend to merge, forming either incomplete or complete annular scotomas [54], making a differential diagnosis with PPACD cases difficult.

In SD-OCT of sector RP, a correlation between the external limiting membrane (ELM) and ellipsoid zone (EZ) integrity and the hyper-autofluorescent bands identified on FAF has been described [19,54,59]. In contrast, areas with retinal thinning, particularly where the OS of the photoreceptors and the outer nuclear layer are no longer present, are typically associated with decreased autofluorescence signals [55]. In cases of sector RP, SDOCT typically reveals initial pathological changes characterized by the disruption of the interdigitation zone, progressing to the EZ band, and ultimately affecting the ELM [54]. As RP advances, these structural alterations culminate in total deterioration of the OS of photoreceptors and the outer nuclear layer. Interestingly, inner retinal layers often remain structurally intact and may exhibit paradoxical thickening, potentially attributable to compensatory mechanisms such as retinal nerve fiber layer edema or neuroglial remodeling caused by atrophy of the outer retinal layers [19,26,54,59].

FAF imaging in sector RP reveals increased autofluorescence in a foveal ring or arc pattern, which is not evident on fundus examination, detectable in over half of RP patients [54]. The hyperautofluorescent ring observed in RP corresponds to a zone of altered photoreceptor OS and increased lipofuscin accumulation, often paralleled by progressive retinal atrophy and EZ disruption near the ring’s internal margin. The diameter of this ring typically contracts gradually over time [54]. Consistent with the progression of RP, FAF imaging in adult patients frequently reveals diminished or patchy autofluorescence in the mid-periphery, indicative of the corresponding peripheral visual field loss [54].

Generally, ERG responses are pivotal in diagnosing RP, particularly in the early stages of the disease. Full-field ERG reveals a significant reduction in a- and b-wave amplitudes, having a greater scotopic involvement [19]. However, in instances where retinal involvement is localized or incomplete, ERG findings can present as normal. Such cases often exhibit a diminished maximum ERG response amplitude, reflecting the localized nature of the disorder. These ERG alterations can be similar to those recorded in PPACD patients [1,3,5,6,55].

The genetic spectrum of RP is vast and continues to grow with new gene discoveries. The intricate relationship between certain genes implicated in RP and their role in rod photoreceptor function suggests a potential avenue for further investigation, particularly in connection with PPACD, a condition also affecting mainly these photoreceptors. Intriguingly, the mutations identified in the case report on PPACD by Tabbaa and colleagues [3], such as NR2E3, PCARE, and RPGRIP1, show a genetic overlap with RP, indicating shared molecular pathways that warrant further exploration. However, these variants were identified in PPHCD, which, although deemed a different phenotypic trait of PPACD, leaves the potential genetic involvement of PPACD unresolved.

Notably, most genetic variations associated with RP impact the rod photoreceptors, which are probably also the primary cells affected in PPACD. The genetic variants leading to photoreceptor degeneration merit further investigation in the context of PPACD to enhance our understanding and approach to this condition [57,60].

### 4.2. Diagnostic Differences between Choroideremia and PPACD

Choroideremia (CHM) is an X-linked hereditary choroidal dystrophy with a prevalence of 1 in 50,000 patients [61]. The condition is marked by progressive degeneration of the choriocapillaris, RPE, and retina. This disease primarily affects males due to its genetic inheritance pattern [61,62]. In its early stages, CHM presents a patchy depigmentation in the mid-peripheral retina, described as “salt and pepper” mottling, the lesions gradually move centripetally sharing the same topographical locations as the atrophic areas in PPACD but in a more patchy distribution [61,62,63]. The terminal phase of CHM shares similarities with the end stage of PPACD, characterized by extensive retinal atrophy. The late-stage presentations underscore the complex and overlapping phenotypes of retinal dystrophies, necessitating a thorough understanding of the genetic and clinical nuances to ensure accurate differential diagnosis and management.

CHM is caused by mutations in the CHM gene, which encodes the Rab escort protein-1 (REP-1). REP-1 is essential for intracellular interactions, specifically in directing Rab proteins to membranes of various cell organelles, and its deficiency leads to premature cell death [61,63]. However, due to the presence of REP-2, encoded by the CHML gene, most human cells can compensate for the lack of REP-1. This compensation does not extend to RPE and photoreceptor cells, which is why these cells are specifically affected in CHM [61,64].

The genetic mutations associated with CHM are predominantly loss-of-function mutations, with over 346 identified variations that lead to a broad spectrum of clinical manifestations. Patients affected experience nyctalopia in their early years, which also represents the classic symptom of PPACD, progressing to significant peripheral vision loss over time [62,64].

Considering the phenotypic parallels observed between PPACD and CHM, an accurate multimodal examination and a genetic background assessment for CHM can help in differential diagnosis, to investigate whether a genetic overlap may exist between these entities. 

### 4.3. Diagnostic Differences between Gyrate Atrophy of the Choroid and Retina and PPACD

Gyrate Atrophy of the choroid and retina (GACR) can share similar pathological alterations with PPACD, consisting of a complete absence of RPE and choriocapillaris [1,3,5,6,65,66]. Regarding the topographical localization of the lesions, in PPACD the peripheral retina and macular region are usually intact, while GACR presents with typical mid-peripheral lesions that coalesce and advance centripetally towards the posterior pole. Therefore, a macular involvement is characteristic of the late phases of the latter disease [67]. Furthermore, while cystoid macular edema is a rare occurrence in PPACD [5], it is frequent in the late phases of GACR [68]. In GACR, a central vision loss secondary to cystoid macular edema is typical in the late phases of the disease, in contrast to the peripheral vision loss seen in PPACD [65,66,68].

GACR is a condition linked to diverse mutations in the mitochondrial enzyme ornithine-delta-aminotransferase (OAT) gene located on chromosome 10q26 [66,68]. This enzyme, which requires pyridoxal 5′phosphate (PLP)—a vitamin B6 derivative—as a cofactor, is crucial in the degradation pathway of ornithine in humans. The disorder follows an autosomal recessive pattern of inheritance [68]. To date, over fifty variants in the OAT gene have been discovered, with missense mutations being the most common [69]. These mutations often result in a truncated form of the OAT, leading to its rapid degradation and subsequent metabolic disruptions [70,71]. Despite the clinical similarities, PPACD and GACR seem to present a completely different genetic substrate. Genetic investigations and ornithine plasmatic levels can be decisive in the differential diagnosis between the two pathologies, whenever the clinical and diagnostic setting could appear equivocal.

### 4.4. Diagnostic Differences between Central Areolar Choroidal Dystrophy and PPACD

Central areolar choroidal dystrophy (CACD) is characterized by a noticeable absence of the RPE, choriocapillaris, and neurosensory retina in the regions affected, revealing an atrophic choroid with prominent choroidal vessels overlying the exposed sclera [72,73]. Similar to PPACD, the optic nerve, retinal vasculature, and peripheral retina remain intact [1,2,3,4,72]. Unlike PPACD, CACD has a significant impact on the macula, with lesions ranging from hypopigmentation to complete atrophy, typically presenting bilaterally and symmetrically within the macular region. This macular involvement results in distinct clinical symptoms, including bilateral central scotoma or bilateral vision loss, while the peripheral visual field generally remains unaltered [52,74].

CACD predominantly follows an autosomal dominant inheritance pattern, although autosomal recessive and sporadic cases have been documented [75]. Variations in six genes (PRPH2, GUCA1A, GUCY2D, CDHR1, ABCA4, and TTLL5) have been identified in association with the dominant monogenic forms of CACD. Of note, mutations in the NR2E3 gene, which is situated at 15q23 and encodes a photoreceptor-specific nuclear receptor, have been implicated in both CACD and PPACD, highlighting a possible shared genetic pathway in the pathogenesis of these conditions [52]. The common mutation of the NR2E3 gene suggests the crucial role of rod differentiation in the development and evolution of both pathologies, suggesting possible common genetic therapeutic targets.

## 5. Conclusions

The present review explored the existing literature on PPACD, elucidating the clinical and multimodal imaging features and the potential genetic variants that may be involved in disease pathogenesis. This rare clinical entity presents in mid-aged individuals with a normal or subnormal visual acuity with nyctalopia as the initial presentation. Clinically, the lesions configure a characteristic annular pattern involving the peripapillary region and extend toward the retinal vascular arcades. The topographic distribution of the lesions characteristically spares the macular region and the peripheral retina without arteriolar attenuation or waxy pallor of the optic disc, helping the distinction with other inherited retinal dystrophies. As the disease progresses, peripheral vision tends to be more affected leading to peripheral visual depression.

The present work delineates clinical and genetic parallels with other inherited retinal diseases to clarify the pathogenic pathway. In PPACD, some features like the age of presentation, clinical appearance, symptoms like nyctalopia and peripheral vision loss, and the predominant photoreceptor involvement suggest possible genetic variants involved in the pathogenesis similar to other inherited retinal dystrophies. Despite this, only a few variants of uncertain significance were identified in a phenotypic variant of PPACD known as PPHCD. The most interesting variants implicate NR2E3, PCARE, and RPGRIP1 genes that are involved in retinal homeostasis. Through an in-depth analysis of multimodal imaging and genetic testing, we consolidated the diagnostic criteria that differentiate PPACD from phenotypically similar conditions, which is crucial for accurate clinical management and genetic counseling. To date, the genetic underpinnings of PPACD remain enigmatic, warranting further investigation into the molecular mechanisms driving its development. In this regard, the synthesis of the current literature has provided a foundation for future inquiries, suggesting that a deeper exploration of the genetic variations associated with PPACD could shed light on novel therapeutic targets.

Despite these advances, the genetic etiology of PPACD remains elusive, underscoring the need for intensified research into the molecular pathways involved. While the present synthesis of the literature lays the groundwork for future research, it also highlights significant limitations. The scarcity of large-scale genetic studies and the complexity of phenotypic variability present challenges to pinpointing specific genetic markers. Furthermore, the heterogeneity in clinical presentations of PPACD suggests that multiple genes may be implicated, necessitating a broader approach to genetic analysis. The application of advanced genetic sequencing techniques and bioinformatics is crucial to unravel the etiological nature of PPACD.

Such efforts will not only advance our scientific understanding but also potentially uncover new avenues for therapeutic intervention. Recognizing these limitations points to the necessity for a multidisciplinary approach that integrates ophthalmic imaging, genetic analysis, and functional studies to holistically address the gaps in our current knowledge about this perplexing condition.

## Figures and Tables

**Table 1:** Summary of posterior polar annular choroidal dystrophy (PPACD) appearance with different imaging modalities.

Imaging Modality	PPACD Appearance
**Fundus Examination**	Peripapillary chorioretinal atrophy reaches out towards the temporal vascular arcade, creating an annular pattern while leaving the foveal area unaffected asymmetricity is often found [1,2,3,5,6,10].
**FAF**	Annular pattern of hypoautofluorescence in a peripapillary distribution, extending in a concentric pattern to the temporal arcades that corresponds to the area of atrophy of the chorioretina. A perifoveal hyperautofluorescent border frequently demarcates the transition from healthy to atrophic retinal tissue [1,2,3,5,10].
**FA**	Window defect with visualization of the underlying choroidal vasculature attributable to an absence of perfusion in the retinal and choroidal vessels, along with staining in the late frames in a concentric configuration surrounding the optic disc and central macula. Areas of late leakage surrounding the fovea in one case [3].
**SD-OCT**	Thinning of the outer retina and choriocapillaris in regions exhibiting atrophic changes. Additionally, disturbances in the organization of the outer retinal structures and alterations in the EZ have been observed [3]. Involvement of the parafovea with parafoveal cystic intraretinal fluid was reported in one case [3]. Only one case [5] presented cystoid macular edema and outer retinal atrophy leaving the subfoveal area unaffected.
**SS-OCT**	Physiological foveal and subfoveal choroid, with marked thinning of the retina with complete disorganization of retinal layers in the atrophic chorioretinal area. In the atrophic regions, there is typically a notable decrease in the thickness of the choroid, characterized by the absence of Sattler’s layer and diminished choroidal vascularity [1,2,6].
**OCTA**	In the context of retinal atrophy, imaging often reveals the preservation of normal vascularization within the foveal and subfoveal choroidal regions. Conversely, there is a notable diminution in the depth of the capillary plexus, along with an evident depletion of the choriocapillaris corresponding to the atrophic zones observed in both eyes [5,6].
**Perimetric Testing**	Two cases [2,3] showed superior arcuate scotomas in botheyes. In the two cases reported by Lenis and colleagues, the authors reported enlarged blind spots in perimetric testing correlating with the pattern of peripapillary atrophy [10]. In the case reported by Narayanan and colleagues in 2019, perimetric testing demonstrated generalized depressed points, predominantly in the pericentral area [1].
**ERG**	Predominately depressed cone responses with normal rod responses, and not only in the atrophy region. In the case reported by Narayanan and colleagues in 2019, each eye exhibited a diminished response in both scotopic and photopic a-wave and b-wave amplitudes, alongside a decrease in the 30 Hz flicker response and oscillatory potentials, coupled with a prolongation of implicit times [1]. Sone and colleagues reported a case of mild reduction of a-wave amplitudes in scotopic and combined rod–cone responses in both eyes.
**LSFG**	LSFG was conducted in only one case and it showed diminished warmth in the coloration at the posterior pole in both eyes [2].

FAF: Fundus Autofluorescence; FA: Fluorescein Angiography; SDOCT: Spectral-domain Optical Coherence Tomography; EZ: Ellipsoid Zone; SSOCT: Swept-source Optical Coherence Tomography; OCTA: Optical Coherence Tomography Angiography; ERG: Full-field Electroretinogram; LSFG: Laser speckle flowgraphy.

**Table 2:** Summary of retinal genes: functional roles and chromosomal locations.

Gene	Biological Role	Associated Retinal Processes	Chromosomal Location
ABCA4	ATP-binding cassette transporter [28,29,30,31,32,33]	Protein involved in the visual cycle of photoreceptors [28,29,30,31,32,33]	1p22.1 [28,29,32]
BEST1	Integral membrane protein [34]	Involved in ion transport across RPE [34]	11q13 [34]
PRPH2	Cell-surface protein [35]	Plays a role in the visual phototransduction process and in the structural integrity of photoreceptor outer segments [35]	6p21.1 [35]
CDH3	Cell–cell adhesion glycoprotein [36,37]	Cadherin-related protein, may play a role in cell adhesion [36,37]	16q22.1 [36,37]
EFEMP1	Extracellular matrix protein [38]	Associated with the extracellular matrix and may influence eye development [38]	2p16.1 [38]
ELOVL4	Membrane-bound protein [39,40]	Involved in the elongation of very long-chain fatty acids [39,40,41]	6q14.1 [39,40,41]
IMPG1	Matrix proteoglycan [42,43,44]	Plays a role in the homeostasis and function of the retinal pigment epithelium [42,43,44]	6q14.2 [42,43,44]
IMPG2	Matrix proteoglycan [45,46,47]	Involved in the structural integrity of the photoreceptor outer segment [45,46,47]	3q12.3 [45,46,47]
PROM1	Transmembrane protein [48,49]	Involved in photoreceptor disk morphogenesis [48,49]	4p15.32 [48,49]
TIMP3	Metallopeptidase inhibitor [50]	Involved in ocular development [50]	22q12.3 [50]
CCD2D2A	Binding domain protein [3]	Uncertain significance, possibly related to cilia structure and function [3]	4q21.1 [3]
CEP78	Centrosomal protein [51]	May be involved in ciliary functions [51]	9q21.13 [51]
NR2E3	Nuclear receptor [3,21,22,52]	Key transcription factor for photoreceptor development [3,21,22,52]	15q23 [21,22]
PCARE	Ciliary and actin-associated protein [3,23,53]	Probably associated with the primary cilium of the outer segment [3,23,53]	2p23.2 [53]

## Data Availability

No new data were created or analyzed in this study. Data sharing is not applicable to this article.
